# Structure of BT_3984, a member of the SusD/RagB family of nutrient-binding molecules

**DOI:** 10.1107/S1744309110032999

**Published:** 2010-09-22

**Authors:** Constantina Bakolitsa, Qingping Xu, Christopher L. Rife, Polat Abdubek, Tamara Astakhova, Herbert L. Axelrod, Dennis Carlton, Connie Chen, Hsiu-Ju Chiu, Thomas Clayton, Debanu Das, Marc C. Deller, Lian Duan, Kyle Ellrott, Carol L. Farr, Julie Feuerhelm, Joanna C. Grant, Anna Grzechnik, Gye Won Han, Lukasz Jaroszewski, Kevin K. Jin, Heath E. Klock, Mark W. Knuth, Piotr Kozbial, S. Sri Krishna, Abhinav Kumar, Winnie W. Lam, David Marciano, Daniel McMullan, Mitchell D. Miller, Andrew T. Morse, Edward Nigoghossian, Amanda Nopakun, Linda Okach, Christina Puckett, Ron Reyes, Henry J. Tien, Christine B. Trame, Henry van den Bedem, Dana Weekes, Keith O. Hodgson, John Wooley, Marc-André Elsliger, Ashley M. Deacon, Adam Godzik, Scott A. Lesley, Ian A. Wilson

**Affiliations:** a Joint Center for Structural Genomics, http://www.jcsg.org, USA; b Program on Bioinformatics and Systems Biology, Sanford–Burnham Medical Research Institute, La Jolla, CA, USA; c Stanford Synchrotron Radiation Lightsource, SLAC National Accelerator Laboratory, Menlo Park, CA, USA; d Protein Sciences Department, Genomics Institute of the Novartis Research Foundation, San Diego, CA, USA; e Center for Research in Biological Systems, University of California, San Diego, La Jolla, CA, USA; f Department of Molecular Biology, The Scripps Research Institute, La Jolla, CA, USA; g Photon Science, SLAC National Accelerator Laboratory, Menlo Park, CA, USA

**Keywords:** structural genomics, starch-utilization system, gut microbiome, metagenomics

## Abstract

The crystal structure of the *Bacteroides thetaiotaomicron* protein BT_3984 was determined to a resolution of 1.7 Å and was the first structure to be determined from the extensive SusD family of polysaccharide-binding proteins. SusD is an essential component of the *sus* operon that defines the paradigm for glycan utilization in dominant members of the human gut microbiota. Structural analysis of BT_3984 revealed an N-terminal region containing several tetratricopeptide repeats (TPRs), while the signature C-terminal region is less structured and contains extensive loop regions. Sequence and structure analysis of BT_3984 suggests the presence of binding interfaces for other proteins from the polysaccharide-utilization complex.

## Abstract 2

The crystal structure of BT_3984, a SusD-family protein, reveals a TPR N-terminal region providing support for a loop-rich C-terminal subdomain and suggests possible interfaces involved in sus complex formation.

## 1. Introduction

The microbiota that inhabit the mammalian distal gut are capable of foraging on a wide variety of dietary and host carbohydrates. In *Bacteroides*, the dominant bacterial phylum in the mammalian gut, this process involves the deployment of close to 100 polysaccharide-utilization loci (PUL; Martens *et al.*, 2009 ▶). The starch-utilization system (sus) of *B. thetaiotaomicron* is the prototypic and best studied PUL (Tancula *et al.*, 1992 ▶). The *sus* operon consists of eight genes that code for seven proteins involved in starch binding (SusC–F) and hydrolysis (SusA, SusB and SusG) and the maltose-activated transcriptional regulator SusR. SusA and SusB are located in the periplasm and SusG is located on the outer membrane. SusCDEFG are likely to form a complex that binds, processes and imports starch (Koropatkin & Smith, 2010 ▶). SusD, in association with SusC, a predicted TonB-dependent β-barrel porin, constitutes the minimal starch-binding unit, with further binding affinity provided by SusE and SusF and starch hydrolysis by the α-amylase SusG (for a review, see Martens *et al.*, 2009 ▶).

We have determined the structure of a SusD homolog, the BT_3984 protein from *B. thetaiotaomicron* VPI-5482, which is a prominent member of the human gut microbiome, using the semiautomated high-throughput pipeline of the Joint Center for Structural Genomics (JCSG; Lesley *et al.*, 2002 ▶) as part of the NIGMS Protein Structure Initiative (PSI). The BT_3984 protein has a molecular weight of 57 kDa (residues 1–515) and a calculated isoelectric point of 4.9. At the time of deposition, BT_3984 was the first structural representative of the PF07980 Pfam family of SusD/RagB homologs and belongs to a PUL with unknown specificity and very limited similarity to the archetypal *sus* operon. This locus contains a SusC homolog (BT_3983) but no SusE or SusF homologs. Two glycosyl hydrolases are present in this operon, but are not homologous to those in the *sus* operon. Additional structures of proteins from the SusD family have since been determined by the JCSG and other groups in an attempt to uncover the structural determinants of starch recognition by this family. Moreover, several of these structures have been determined with a variety of bound ligands, with the results suggesting a combination of shape-specific, composition-specific and avidity mechanisms (Koropatkin *et al.*, 2008 ▶, 2009 ▶). Although a direct interaction between SusD and SusC has been demonstrated by limited proteolysis and cross-linking experiments (Cho & Salyers, 2001 ▶), the interacting surface has not been mapped on SusD.

Structural analysis of BT_3984 revealed two tightly packed sub­domains. The N-terminal region consists of three typical tetratrico­peptide repeats (TPRs) that form an αα right-handed superhelix. A mostly unstructured region of ∼100 residues separates the N-­terminal TPRs from a fourth TPR located in the C-terminal region that continues the superhelix. Additional elements, including helices and long loops, define the C-terminal subdomain, which has been characterized as structurally unique. Structure comparison between BT_3984 and other SusD homologs offers insights into the minimal starch-binding unit in this family.

## 2. Materials and methods

### 2.1. Protein production and crystallization

Clones were generated using the Polymerase Incomplete Primer Extension (PIPE) cloning method (Klock *et al.*, 2008 ▶). The gene encoding BT_3984 (GenBank NP_812895; Swiss-Prot Q8A0N7) was amplified by polymerase chain reaction (PCR) from *B. thetaiota­omicron* VPI-5482 genomic DNA using *PfuTurbo* DNA polymerase (Stratagene) and I-PIPE (Insert) primers (forward primer, 5′-ctg­tacttccagggcAACTATGAGAATATCAATTCCAACCC-3′; reverse primer, 5′-aattaagtcgcgttaTTTTTTAGAAGCCCACCATACATCT­G-3′; target sequence in upper case) that included sequences for the predicted 5′ and 3′ ends. The expression vector pSpeedET, which encodes an amino-terminal tobacco etch virus (TEV) protease-cleavable expression and purification tag (MGSDKIHHHHHHENLYFQ/G), was PCR-amplified with V-PIPE (Vector) primers. The V-­PIPE and I-PIPE PCR products were mixed to anneal the amplified DNA fragments together. *Escherichia coli* GeneHogs (Invitro­gen) competent cells were transformed with the V-PIPE/I-PIPE mixture and dispensed onto selective LB–agar plates. The cloning junctions were confirmed by DNA sequencing. Using the PIPE method, the section of the gene encoding residues 1–22 was deleted as it was predicted to code for a signal peptide. Expression was performed in selenomethionine-containing medium with suppression of normal methionine synthesis. At the end of fermentation, lysozyme was added to the culture to a final concentration of 250 µg ml^−1^ and the cells were harvested and frozen. After one freeze–thaw cycle, the cells were homogenized in lysis buffer [50 m*M* HEPES pH 8.0, 50 m*M* NaCl, 10 m*M* imidazole, 1 m*M* tris(2-carboxyethyl)phos­phine–HCl (TCEP)] and passed through a Microfluidizer (Microfluidics). The lysate was clarified by centrifugation at 32 500*g* for 30 min and loaded onto nickel-chelating resin (GE Healthcare) pre-equilibrated with lysis buffer; the resin was washed with wash buffer [50 m*M* HEPES pH 8.0, 300 m*M* NaCl, 40 m*M* imidazole, 10%(*v*/*v*) glycerol, 1 m*M* TCEP] and the protein was eluted with elution buffer [20 m*M* HEPES pH 8.0, 300 m*M* imidazole, 10%(*v*/*v*) glycerol, 1 m*M* TCEP]. The eluate was buffer-exchanged with TEV buffer (20 m*M* HEPES pH 8.0, 200 m*M* NaCl, 40 m*M* imidazole, 1 m*M* TCEP) using a PD-10 column (GE Healthcare) and incubated with 1 mg TEV protease per 15 mg eluted protein. The protease-treated eluate was run over nickel-chelating resin (GE Healthcare) pre-equilibrated with HEPES crystallization buffer (20 m*M* HEPES pH 8.0, 200 m*M* NaCl, 40 m*M* imidazole, 1 m*M* TCEP) and the resin was washed with the same buffer. The flowthrough and wash fractions were combined and concentrated to 18.6 mg ml^−1^ by centrifugal ultrafiltration (Millipore) for crystallization trials. BT_3984 was crystallized using the nanodroplet vapor-diffusion method (Santarsiero *et al.*, 2002 ▶) with standard JCSG crystallization protocols (Lesley *et al.*, 2002 ▶). Sitting drops composed of 200 nl protein solution mixed with 200 nl crystallization solution were equilibrated against a 50 µl reservoir at 277 K. The crystallization reagent that produced the BT_3984 crystal used for structure determination was composed of 0.2 *M* ammonium acetate, 30% PEG 4000 and 0.1 *M* citrate pH 5.6. A plate-like crystal of approximate dimensions 100 × 60 × 20 µm was harvested after 14 d at 277 K for data collection. No further cryoprotectant was required. Initial screening for diffraction was carried out using the Stanford Automated Mounting system (SAM; Cohen *et al.*, 2002 ▶) at the Stanford Synchrotron Radiation Lightsource (SSRL, Menlo Park, California, USA). The diffraction data were indexed in space group *C*222_1_. The oligomeric state of BT_3984 in solution was determined using a 1 × 30 cm Superdex 200 size-exclusion column (GE Healthcare) coupled with miniDAWN static light-scattering (SEC/SLS) and Optilab differential refractive-index detectors (Wyatt Technology). The mobile phase consisted of 20 m*M* Tris pH 8.0, 150 m*M* NaCl and 0.02%(*w*/*v*) sodium azide. The molecular weight was calculated using *ASTRA* v.5.1.5 software (Wyatt Technology).

### 2.2. Data collection, structure solution and refinement

Multiple-wavelength anomalous diffraction (MAD) data were collected on beamline BL9-2 at the SSRL at wavelengths corresponding to the remote (λ_1_), peak (λ_2_) and inflection point (λ_3_) of a selenium MAD experiment. The remote and inflection-point data were collected interleaved in the first pass with a wedge size of 10°, followed by the peak data. The data sets were collected at 100 K on a MAR Mosaic 325 mm CCD detector (Rayonix, Evanston, Illinois, USA) using the *Blu-Ice* data-collection environment (McPhillips *et al.*, 2002 ▶). The MAD data were integrated and reduced using *XDS* and then scaled with the program *XSCALE* (Kabsch, 1993 ▶, 2010*a* ▶,*b* ▶). Initial substructure solution was performed with *SHELX* (Sheldrick, 2008 ▶) and the phases were refined with *autoSHARP* (Bricogne *et al.*, 2003 ▶; mean figure of merit of 0.59 with nine selenium sites). Density modification with *DM* (Cowtan & Main, 1996 ▶) was followed by automated model building using *ARP*/*wARP* (Cohen *et al.*, 2004 ▶). Model completion and refinement were performed with *Coot* (Emsley & Cowtan, 2004 ▶) and *REFMAC*5.2 (Winn *et al.*, 2003 ▶) using the remote (λ_1_) data. The refinement included experimental phase restraints in the form of Hendrickson–Lattman coefficients and TLS refinement with one TLS group per chain. Data-collection and refinement statistics are summarized in Table 1 ▶.

### 2.3. Validation and deposition

**Figure 1:**
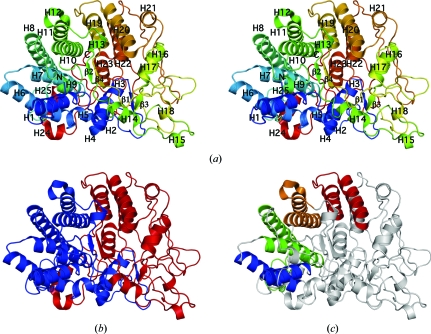

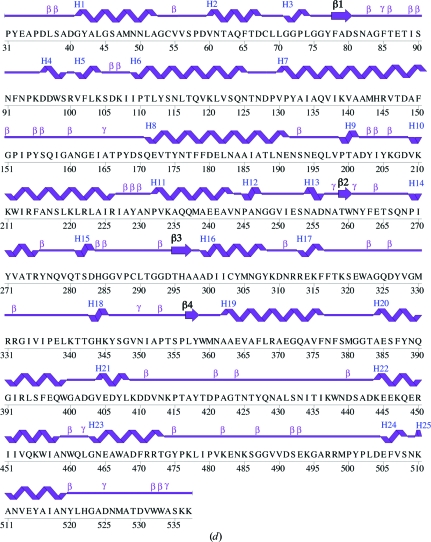
Crystal structure of BT_3984 from *B. thetaiotaomicron* VPI-5482. (*a*) Stereo ribbon diagram of the BT_3984 monomer color-coded from the N-terminus (blue) to the C-­terminus (red). Helices H1–H25 and β-strands (β1–β4) are indicated. (*b*) Ribbon diagram in the same orientation as (*a*) showing the two subdomains of BT-3984 colored in blue and red for the N- and C-terminal regions, respectively. (*c*) Ribbon diagram in the same orientation as in (*a*) showing the four TPRs present in BT_3984: from the N- to C-terminus, TPR1 (blue), TPR2 (green), TPR3 (orange) and TPR4 (red). (*d*) Diagram showing the secondary-structure elements of BT_3984 superimposed on its primary sequence. The labeling of secondary-structure elements is in accord with *PDBsum* (http://www.ebi.ac.uk/pdbsum), where α-helices are labeled H1, H2, H3 *etc*., β-strands are labeled and β-turns and γ-turns are designated by their respective Greek letters (β, γ). For BT_3984, the α-helices (H1–H8, H10–H11, H14, H16–H17 and H19–H25), 3_10_-­helices (H9, H12–H13, H15 and H18) and β-strands (β1–4) are indicated.

The quality of the crystal structure was analyzed using the *JCSG Quality Control* server (http://smb.slac.stanford.edu/jcsg/QC). This server processes the coordinates and data through a variety of validation tools including *AutoDepInputTool* (Yang *et al.*, 2004 ▶), *MolProbity* (Chen *et al.*, 2010 ▶), *WHAT IF* 5.0 (Vriend, 1990 ▶), *RESOLVE* (Terwilliger, 2003 ▶) and *MOLEMAN*2 (Kleywegt, 2000 ▶), as well as several in-house scripts, and summarizes the output. Fig. 1 ▶(*d*) was adapted from an analysis using *PDBsum* (Laskowski *et al.*, 2005 ▶) and all other figures were prepared with *PyMOL* (DeLano Scientific). Atomic coordinates and experimental structure factors for BT_3984 from *B. thetaiotaomicron* VPI-5482 at 1.7 Å resolution have been deposited in the PDB (http://www.pdb.org) and are accessible under code 3cgh.

## 3. Results and discussion

### 3.1. Overall structure

The crystal structure of BT_3984 (Fig. 1 ▶ *a*) was determined to 1.7 Å resolution using the MAD method. Data-collection, model and refinement statistics are summarized in Table 1 ▶. The final model includes one BT_3984 molecule (residues 31–537), one acetate molecule, one zinc ion and 705 water molecules in the asymmetric unit. The partially occupied zinc was tentatively assigned based on electron density, coordination geometry and an X-ray fluorescence excitation scan that showed a small peak above background only for zinc. However, given the low signal-to-background ratio, we could not confirm that the bound ion actually is zinc. Gly0 (which remained at the N-terminus after cleavage of the expression/purification tag), Asn23, Tyr24, Glu25, Asn26, Ile27, Asn28, Ser29 and Asn30 were disordered and were not modeled. The side chains of Glu33, Gln280, Lys309, Lys340, Lys409 and Lys444 were only partially modeled owing to poor or incomplete electron density. The Matthews coefficient (*V*_M_; Matthews, 1968 ▶) is 2.4 Å^3^ Da^−1^ and the estimated solvent content is 48%. The Ramachandran plot produced by *MolProbity* (Chen *et al.*, 2010 ▶) shows that 89.4% of the residues are in favored regions, with no outliers.

BT_3984 is a member of the Pfam SusD/RagB family (PF07980), which includes starch-utilization protein D (SusD) and the immuno­dominant antigen RagB from *Porphyromonas gingivalis* (note that previous releases of Pfam mistakenly included a reference to a human Ras-related GTP-binding RagB protein that was corrected in the Pfam v.24 release). The signature sequence of this Pfam family covers most of the BT_3984 C-terminal region (residues 233–492), a region described by SCOP (http://scop.mrc-lmb.cam.ac.uk/scop/data/scop.b.b.cda.bb.g.b.html) as structurally unique.

The first 20 N-terminal residues of full-length BT_3984 are predicted to form an α-helix (Cole *et al.*, 2008 ▶) that contains a non­cleavable signal sequence (Bendtsen *et al.*, 2004 ▶) that is thought to be responsible for the localization and anchoring of SusD homologs in the outer membrane. A calculation of the hydrophobic moment (Rice *et al.*, 2000 ▶) of this helix, according to Eisenberg, Weiss *et al.* (1984 ▶), reveals a small hydrophobic moment (maximum value 0.4) and strong hydrophobicity (GRAVY index of 0.98) that is typical of monomeric transmembrane anchors (Eisenberg, Schwarz *et al.*, 1984 ▶). Therefore, to improve protein solubility and increase the likelihood of crystallization, this region was excluded from the expression construct.

**Figure 2:**
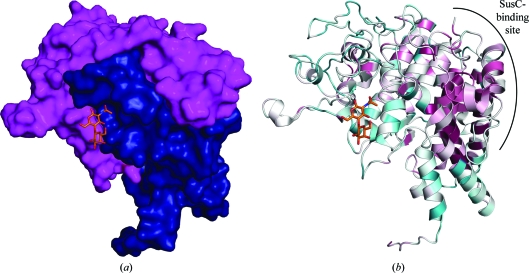
Structural organization of BT_3984 and homologs. (*a*) Surface representation of BT_3984 showing the two interlocking regions (N-terminal subdomain, residues 31–265, in blue; C-terminal subdomain, residues 266–537, in magenta) with the binding site for *N*-acetyllactosamine (LacNAc, in orange ball-and-stick representation) lying across the domain interface. LacNAc was modeled from structural superposition of BT_3984 (PDB code 3cgh; residues 31–537) with another SusD homolog, BT_1043 (PDB code 3ehn; residues 33–546). (*b*) Ribbon diagram of BT_3984 in the same orientation as in (*a*) colored by sequence conservation according to *ConSurf* (Landau *et al.*, 2005 ▶). High conservation among BT_3984 homologs is indicated in maroon and low conservation is indicated in turquoise. The potential SusC-binding interface is indicated.

BT_3984 adopts a compact globular structure that at first glance resembles a single-domain protein (Fig. 1 ▶ *a*). However, some domain-prediction servers detect a two-domain arrangement, with the N-­terminal subdomain ending with the first two TPRs (Cheng, 2007 ▶). Thus, taking into account both structural and sequence-conservation features of the protein (see below), we subdivided BT_3984 into a highly conserved and more structured N-terminal region (residues 31–265) and a more ‘flexible’, loop-rich and less conserved C-terminal region (residues 266–552) (Fig. 1 ▶ *b*). The N-terminal subdomain is characterized by a tetratricopeptide repeat-like right-handed superhelix containing three tetratricopeptide repeats (TPRs; helices H1 and H6 for TPR1, H7–H8 for TPR2 and H10–H11 for TPR3; Figs. 1 ▶ *c* and 1 ▶ *d*). The C-terminal subdomain (residues 266–552) is characterized by a fourth TPR (helices H19–H20) and by long unstructured stretches that comprise over half the sequence of this domain. Located in the center of the protein, helices H22 and H23 separate the TPRs from the loop-rich section of the structure. Outside the TPRs, the two subdomains interlock like two hands in a handshake (Fig. 2 ▶ *a*) in an interaction that implicates helices H2–H5 from the N-­terminal region and helices H14, H24 and H25 from the C-terminal subdomain. The region C-terminal to helix H23 extends to the top of the TPR superhelix, contacts all three N-terminal TPRs and inserts between TPR1 and TPR2. Two short antiparallel β-sheets form along the subdomain interface (Fig. 1 ▶ *b*). The first sheet (strands β1–β3) is located proximal to the sugar-binding site (Koropatkin *et al.*, 2008 ▶, 2009 ▶), while the second (strands β2–β4) forms along the center of the TPR superhelix (Figs. 1 ▶ *b* and 1 ▶ *c*). Several SusD homolog structures which were cocrystallized with sugar ligands (Koropatkin *et al.*, 2008 ▶, 2009 ▶) have revealed a sugar-binding site located along this interface. The entire BT_3984 operon is highly upregulated in rich media culture (TVG) and in the mouse distal gut, irrespective of the food source (Martens *et al.*, 2008 ▶), and the predicted sugar-binding site shows a similar overall fold and glycan-binding architecture to other SusD homologs. However, sequence analysis of SusD homologs shows little conservation (Fig. 2 ▶ *b*) and isothermal titration calorimetry studies have indicated that binding to certain oligosaccharides is non-existent (Koropatkin *et al.*, 2008 ▶) or weak (Koropatkin *et al.*, 2009 ▶), suggesting that the SusD cognate ligand is much larger (a starch polymer) and/or other proteins are required to coordinate a multivalent binding.

Analysis of the crystallographic packing of BT_3984 using the *PISA* server (Krissinel & Henrick, 2007 ▶) indicates a dimer as a potential oligomeric form. This crystallographic dimer interface mainly involves loops from the N-terminus, H9–H10, β3–H14 and H18–β4, and buries a surface area of 975 Å^2^ (42%) per molecule. However, analytical size-exclusion chromatography, in combination with static light scattering, indicates that BT_3984 is likely to be a monomer. This result is comparable with oligomerization studies for another SusD homolog that indicate a primarily monomeric state, although minor oligomers have been observed and in at least one case ligand binding (α-cyclodextrin) appears to induce dimerization (Koropatkin *et al.*, 2008 ▶).

### 3.2. Similarity to other proteins

A search with *FATCAT* (Ye & Godzik, 2004 ▶) and *DALI* (Holm & Sander, 1995 ▶) confirmed the strong similarity of BT_3984 to other SusD homologs (PDB code 3gzs, r.m.s.d. of 1.9 Å over 471 residues, 38% identity, Joint Center for Structural Genomics, unpublished work; PDB code 3ehm, r.m.s.d. of 2.2 Å over 475 residues, 27% identity, Koropatkin *et al.*, 2009 ▶; PDB code 3ejn, r.m.s.d. of 3.0 Å over 421 residues, 17% identity, Joint Center for Structural Genomics, unpublished work; PDB code 3fdh, r.m.s.d. of 3.0 Å over 413 residues, 16% identity, Joint Center for Structural Genomics, unpublished work). Other similarities involve different TPR-containing proteins (PDB code 1elr, r.m.s.d. of 2.8 Å over 124 residues, 15% identity; Scheufler *et al.*, 2000 ▶) and 14-3-3 proteins (PDB code 2ijp, r.m.s.d. of 4.1 Å over 168 residues, 5% identity; Structural Genomics Consortium, unpublished work).

TPRs are degenerate 34-amino-acid repeated motifs that have been identified in proteins represented in all kingdoms of life. They generally form amphipathic helix pairs that pack at an angle of approximately 24° with respect to one another and mediate protein–protein interactions and multi-protein complex assemblies with other TPR-containing or non-TPR-containing proteins (Lamb *et al.*, 1995 ▶). The similarity between 14-3-3 and TPR proteins has been noted previously (Das *et al.*, 1998 ▶), with 14-3-3 proteins being considered divergent members of the TPR superfamily.

Structure-based alignments have shown that TPR motifs involve a consensus sequence (Trp4–Leu7–Gly8–Tyr11–Ala20–Phe24–Ala27–Pro32) defined by a largely alternating pattern of small and large amino acids (D’Andrea & Regan, 2003 ▶). However, the presence of long loop insertions and the degenerate nature of the TPR signature sequence results in an inability to predict the TPRs in BT_3984 and other SusD homologs through sequence analysis using current algorithms (Koropatkin *et al.*, 2009 ▶).

Comparison of BT_3984 with other SusD and SusD-like structures shows that the most highly conserved regions of SusD in terms of both sequence and structure cluster on one side of the molecule. These conserved regions involve the second half of TPR1 (H6), TPR2, the first half of TPR3 (H10) and the C-terminal helices (H24 and H25) (Fig. 1 ▶). It seems logical to speculate that such highly conserved regions would be involved in interactions with the obligatory binding partner of each SusD homolog, namely the respective SusC homolog, which in the case of BT_3984 is BT_3983. In support of this hypothesis, the *SHARP*2 protein–protein interaction server (Murakami & Jones, 2006 ▶) lists the above-mentioned TPRs as the top hit for both SusD (PDB code 3ckc; Koropatkin *et al.*, 2008 ▶) and BT_3938 (PDB code 3cgh; this work). These are the only conserved secondary-structure elements shared between the two homologs in terms of interfaces that are predicted to interact with another protein (Fig. 2 ▶ *b*).

**Figure 3:**
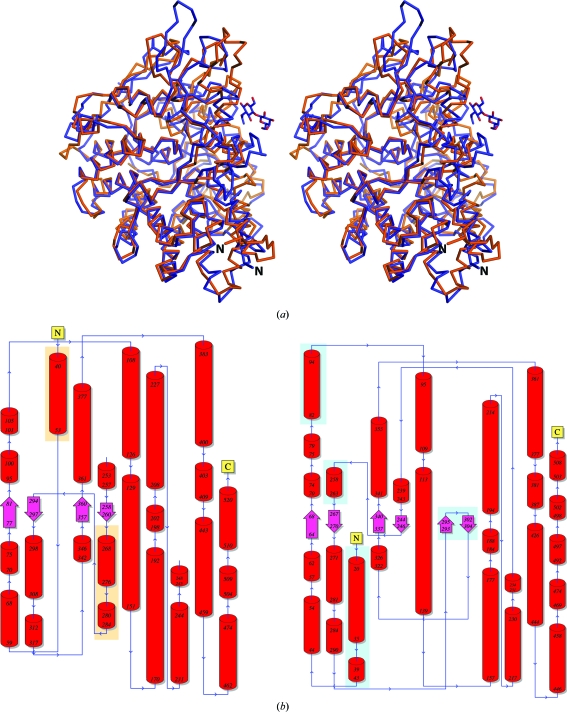
Structure comparison of BT_3984 and BT_1043. (*a*) Stereo ribbon diagram of BT_3984 (PDB code 3cgh; orange) and BT_1043 (PDB code 3ehn; blue). The *N*-­acetyllactosamine sugar cocrystallized with BT_1043 is shown in ball-and-stick representation and the N-terminus of each protein is indicated. (*b*) Topology diagrams of BT_3984 (left) and BT_1043 (right). N-terminal and C-terminal regions and sequence limits for secondary-structure elements are indicated. Secondary-structure elements missing from either structure are indicated by orange- and blue-highlighted boxes for BT_3984 and BT_1043, respectively.

Differences in the TPR orientations between BT_3984 and other published SusD structures (Koropatkin *et al.*, 2008 ▶, 2009 ▶) include changes in length and the presence of kinks in helix H1 (the first half of TPR1), an ∼3 Å shift in the position of helix H11 (the second half of TPR3, with H10 remaining unchanged) and differences in the tilt of helix H20 (the first half of TPR4) (Fig. 3 ▶). By analogy with the conserved TPRs, we propose that these variable TPRs may be involved in binding to more variable members of the SusD-anchored PULs such as, in the case of BT_3984, a predicted uncharacterized glycosyl hydrolase BT_3985.

The SusD protein family (PF07980) contains over 2000 homologs that are predicted to be involved in nutrient binding and whose presence on the outer membrane predisposes them to be effective antigens. The availability of multiple structures of members of this family illustrates the complex evolutionary history of this protein family and specifically the evolution of diverse specificities of *Bacteroides* polysaccharide-ulitization loci. Models of BT_3984 homologs can be accessed at http://www1.jcsg.org/cgi-bin/models/get_mor.pl?key=3cghA.

Additional information about BT_3984 is available from TOPSAN (Krishna *et al.*, 2010 ▶; Weekes *et al.*, 2010 ▶; http://www.topsan.org/explore?PDBid=3cgh).

## 4. Conclusions

The first solved structural representative of the PF07980 family reveals a novel carbohydrate-binding helical fold and suggests interfaces that are implicated in starch-utilization system formation.

## Supplementary Material

PDB reference: BT_3984 from *B. thetaiota­omicron*, 3cgh

## Figures and Tables

**Table 1:** Summary of crystal parameters and data-collection and refinement statistics for BT_3984 (PDB code 3cgh) Values in parentheses are for the highest resolution shell.

	λ_1_ MADSe	λ_2_ MADSe	λ_3_ MADSe
Space group	*C*222_1_		
Unit-cell parameters (Å)	*a* = 49.78, *b* = 125.27, *c* = 174.65		
Data collection			
Wavelength (Å)	0.9184	0.9791	0.9792
Resolution range (Å)	28.0–1.7 (1.76–1.70)	28.1–1.7 (1.76–1.70)	28.0–1.7 (1.76–1.70)
No. of observations	221725	219734	219247
No. of unique reflections	60062	60017	59976
Completeness (%)	98.5 (97.1)	98.1 (97.8)	97.8 (97.3)
Mean *I*/σ(*I*)	10.6 (2.5)	10.1 (2.5)	10.1 (2.5)
*R*_merge_ on *I*† (%)	5.6 (32.8)	5.6 (32.9)	5.5 (32.3)
Model and refinement statistics			
Resolution range (Å)	28.0–1.7		
No. of reflections (total)	60025		
No. of reflections (test set)	3037		
Completeness (%)	99.2		
Data set used in refinement	λ_1_ MADSe		
Cutoff criterion	|*F*| > 0		
*R*_cryst_‡	0.140		
*R*_free_§	0.166		
Stereochemical parameters			
Restraints (r.m.s. observed)			
Bond angles (°)	1.52		
Bond lengths (Å)	0.016		
Average isotropic *B* value (Å^2^)	18.1¶		
ESU†† based on *R*_free_ (Å)	0.083		
No. of protein residues	507		
No. of protein atoms	4035		
No. of waters	704		
No. of other molecules	2 (acetate, zinc)		

† *R*_merge_ = 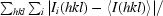 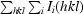.

‡ *R*_cryst_ = 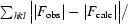 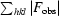, where *F*_calc_ and *F*_obs_ are the calculated and observed structure-factor amplitudes, respectively.

§ *R*_free_ is the same as *R*_cryst_ but for 5.1% of the total reflections that were chosen at random and omitted from refinement.

¶ This value represents the total *B* that includes TLS and residual *B* components.

†† Estimated overall coordinate error (Collaborative Computational Project, Number 4, 1994 ▶; Cruickshank, 1999 ▶).
